# The association between intraocular pressure and different combination of metabolic syndrome components

**DOI:** 10.1186/s12886-016-0263-8

**Published:** 2016-06-06

**Authors:** JunSeok Son, HyunMin Koh, JunHyuk Son

**Affiliations:** Department of Occupational and Environmental Medicine, Samsung Changwon Hospital, Sungkyunkwan University School of Medicine, Changwon, South Korea; Department of Family Medicine, Samsung Changwon Hospital, Sungkyunkwan University School of Medicine, Changwon, South Korea; Department of Ophthalmology, Yeungnam University College of Medicine, #317-1 Daemyung-dong, Nam-gu, Daegu, 705-717 South Korea

**Keywords:** Blood glucose, Blood pressure, High-density lipoproteins, Intraocular pressure, Metabolic syndrome, Triglyceride

## Abstract

**Background:**

Although the association between metabolic syndrome and intraocular pressure is well known, the relationship between the intraocular pressure and different combination of the components of metabolic syndrome has not been actively researched yet. The study aimed to investigate the relationship between the intraocular pressure and metabolic syndrome components with their different combinations.

**Methods:**

Thirty-one thousand two hundred seventy one healthy people aged 19–79 who attended a community hospital for a health check-up between January 2011 and December 2013 were enrolled in the study. Subjects with a history of intraocular disease, at least in one eye and those receiving medical treatment for glaucoma were excluded. Metabolic syndrome was diagnosed following the criteria defined in Circulation 2009.

**Results:**

Subjects with combination of three metabolic syndrome components of triglycerides, abdominal obesity, and fasting glucose had the highest intraocular pressure. And subjects with the combination of four components of blood pressure, high-density lipoproteins, triglycerides, fasting glucose had a significantly higher intraocular pressure than ones with the combination of all five metabolic syndrome components.

**Conclusions:**

The difference in the risk of high intraocular pressure according to the different combination of the metabolic syndrome components could be confirmed. If additional follow-up studies are conducted, the findings can be used as an indicator for predicting intraocular pressure increases in patients with metabolic syndrome.

## Background

Glaucoma is a progressive ophthalmic disease that has the characteristics of optic disc cupping and visual field defects [1]. A family history of glaucoma, high myopia, and high intraocular pressure (IOP) are known as risk factors of glaucoma [2]. Among the various known risk factors, IOP is considered the most important and controllable [3–5].

Although the underlying mechanism of increased IOP is still unclear, a recent study suggested that it might be associated with various health indices, such as hypertension, diabetes, and obesity [6–8]. Reaven [9] indicated that poor health indices, such as abdominal obesity, diabetes, and hypertension, share a single common mechanism that contributes to insulin resistance and glucose intolerance and therefore gave it the name “X syndrome”, which has now been changed to metabolic syndrome (MetS).

The diagnostic criteria for MetS were first announced in 1998 by the World Health Organization (WHO) [10]. Later, the National Cholesterol Education Program-Adults Treatment Panel III of the United States proposed MetS criteria that were more clinically applicable than those announced by the WHO, and these criteria are widely used [11]. Since then, various organizations have proposed slightly different criteria in consideration of ease in diagnosis, and, recently, in 2009, the International Diabetes Federation; National Heart, Lung, and Blood Institute; American Heart Association; World Heart Federation; International Atherosclerosis Society; and International Association for the Study of Obesity reorganized the various criteria for MetS [12]. Although many studies have reported on the association between MetS and IOP, the relationship between MetS components and IOP is not well known. With each component of MetS not independent but correlated, the present study aimed to identify the correlation of IOP with the number and combination of MetS components.

## Methods

### Study subjects

Healthy examinees (31,271) who were between the ages of 19 to 79 and who attended a health promotion center in Changwon region of South Korea, between January 2011 and December 2013 were initially considered as study subjects. Before testing, each participant was interviewed by a trained nurse about previous health problems and medical history, including ocular diseases. A written informed consent was obtained from all participants and participants with a history of intraocular disease or surgery (in at least in one eye), those receiving medical treatment for glaucoma, and those who did not agree to involvement in this study were excluded. Finally, 28,754 healthy examinees were included in the present study.

The study protocol complied with the guidelines of the Declaration of Helsinki and was approved by the Institutional Review Board of Samsung Changwon Hospital, Sungkyunwhan University School of Medicine (2014-SCMC-101-00).

### Study method

#### Questionnaire

A structured questionnaire survey was conducted through one-on-one interviews on the subjects’ medical history, including hypertension, diabetes, dyslipidemia, and ophthalmological history, by surveyors who had received annual standardized training on data collection.

#### Anthropometric measurements

Height was measured to the nearest 0.1 cm by having the subject face forward with their heels and back of their head touching the wall, while weight was measured to the nearest 0.1 kg with an automated scale (GL-150, G-Tech International Co., Ltd., Seoul, Korea) with the subject wearing only light clothing. Waist circumference was measured with the subject positioned in an upright standing position with their feet together, and a tape measure was wrapped around the narrowest parts of the waistline between the ribs and iliac crest. The measurement was taken at the final stage of the breathing cycle without pressing the skin.

#### Clinical examination

Blood pressure (BP) was measured twice with an automatic BP monitor (Jawon Medical Co., Ltd., Seoul, Korea) while the subject was in a sitting position after at least 10 min of being stabilized and the two measured BP values were averaged. If the two measured values showed a discrepancy ≥ 5 mmHg, one additional measurement was taken. The subjects were restricted from smoking or consuming caffeine for 30 min or more prior to the measurements. Blood samples were collected intravenously after 8 h or more of fasting, and a blood test was performed with an enzyme-linked immunosorbent assay to measure triglyceride (TG), high-density lipoprotein cholesterol (HDLC), and fasting glucose (Hitachi Modular DPP, Roche Diagnostics K.K., Tokyo, Japan).

#### Ophthalmologic examination

The IOP was measured when the subject was in a sitting position with noncontact tonometry (Topcon CT60, Topcon Corporation, Tokyo, Japan). The IOP measurements were taken from the central corneal area by an experienced nurse, and the mean value from three measurements was used. All ophthalmological examinations were performed between 8 and 11 am to minimize any effects of diurnal variation, and any IOP ≥22 mmHg was defined as ocular hypertension.

#### Diagnostic criteria of MetS

A diagnosis of MetS required the presence of 3 of the 5 diagnostic criteria of MetS listed below [12]. The criteria for waist circumference was based on the criteria proposed by the Korean Society for the Study of Obesity in 2006, which defined abdominal obesity as a waist circumference of 90 cm or more for males and 85 cm or more for females [13].

① Abdominal obesity: Male waist circumference ≥ 90 cm; Female waist circumference ≥ 85 cm
② Serum TG: ≥150 mg/dL or those taking medication for its treatment
③ HDLC: male < 40 mg/dL, female < 50 mg/dL, or those taking medication for its treatment
④ BP: ≥130/85 mmHg or those taking medication for its treatment
⑤ Fasting glucose: ≥100 mg/dL or those taking medication for its treatment

#### Statistical analysis

In order to examine the general characteristics of the study subjects, an independent t-test was used to compare the mean values of age, waist circumference, systolic and diastolic BP, fasting glucose, lipid profiles, and IOP in the group with IOP ≥22 mmHg and the group with IOP < 22 mmHg. For comparisons of gender, the presence of MetS, and components of MetS, a chi-square test was used for the analysis. For the analysis of the components of MetS in the group with IOP ≥22mmHg and the group with IOP < 22 mmHg, a logistic regression analysis was used to obtain the odds ratio and 95 % confidence interval. All of the statistical analyses were performed with SPSS, ver. 18.0 (IBM Corporation, Armonk, NY, USA).

## Results

### General characteristics of study subjects

**Fig. 1:**
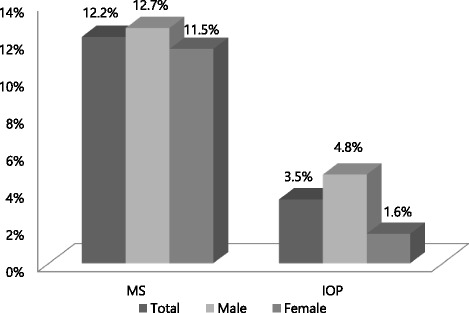
Prevalence of metabolic syndrome and ocular hypertension according to gender. Abbreviations: MetS, Metabolic syndrome; IOP, Intraocular Pressure. *P* < 0.001 Calculated by chi-square test

Among the 28,754 study subjects, there were 16,728 males and 12,026 females, with a mean age of 41.26 years. Three thousand five hundred two out of 28,754 exhibited MetS, which was equivalent to a 12.18 % prevalence rate. With respect to gender, males showed a prevalence rate of 12.65 % (*n* = 2116), while females showed a prevalence rate of 11.53 % (*n* = 1386). And IOP ≥22 mmHg was found in 991 subjects (3.45 %), with 799 males (4.78 %) and 192 females (1.60 %) accounting for the total. A statistically significant difference in both prevalence rate and IOP value were found between males and females (*P* < 0.001) (Fig. 1).

**Fig. 2:**
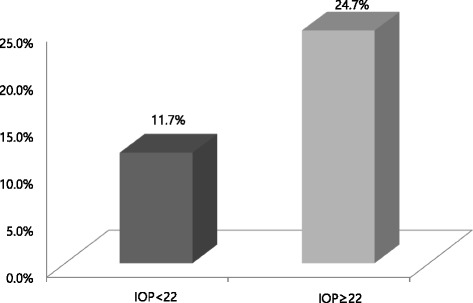
Prevalence of metabolic syndrome according to ocular hypertension. Abbreviation: IOP, Intraocular Pressure. *P* < 0.001 Calculated by chi-square test

The prevalence of MetS in the group with IOP ≥ 22 mmHg and the group with IOP < 22 mmHg was 24.7 and 11.7 %, respectively, which was a significant difference (*P* < 0.001; Fig. 2). Moreover, when each of the mean values for the MetS components was compared between the group with IOP ≥ 22 mmHg and the group with IOP < 22 mmHg, all showed a statistically significant difference (*P* < 0.001; Table 1).

**Table 1:** General characteristics of the study population

	IOP < 22	IOP ≥ 22	P^a^
Age (years)	41.68 ± 7.47	40.83 ± 7.20	<0.001
Waist circumference (cm)	82.47 ± 8.07	86.23 ± 8.45	<0.001
Systolic blood pressure (mmHg)	122.72 ± 16.22	131.42 ± 16.96	<0.001
Diastolic blood pressure (mmHg)	73.18 ± 11.40	78.36 ± 11.90	<0.001
Fasting glucose (mg/dL)	90.05 ± 15.64	96.78 ± 24.35	<0.001
Triglyceride (mg/dL)	110.27 ± 79.44	146.29 ± 105.67	<0.001
HDL cholesterol (mg/dL)	59.06 ± 14.56	55.52 ± 13.61	<0.001
Left IOP (mmHg)	14.72 ± 2.44	22.18 ± 2.00	<0.001
Right IOP (mmHg)	14.74 ± 2.43	22.15 ± 2.12	<0.001

Abbreviations: *IOP* intraocular pressure, *HDL cholesterol* high-density lipoprotein cholesterol

Data are mean ± standard deviation (SD)

^a^Calculated by independent t-test

### Relationship between MetS and IOP

The mean IOP of the right eye in the subjects with MetS showed higher value of 16.05 mmHg than that of 14.85 mmHg in the subjects without MetS, which indicated a statistically significant difference(*P* < 0.001), while the mean IOP value of the left eye was 16.12 and 14.87 mmHg, respectively.

Among the 3502 subjects with characteristics that corresponded to MetS, 73.04 % (*n* = 2558) had three components of MetS, 23.50 % (*n* = 823) had four components, and 3.46 % (*n* = 121) had five components. Moreover, for the cases diagnosed with MetS, the group with IOP ≥22 mmHg had more components of MetS than the group with IOP < 22 mmHg (*P* < 0.001). Having more components of MetS increased the probability of having a higher IOP (*P* for linear trend < 0.001, Table 2).

**Table 2:** Comparisons of intraocular pressures according to metabolic syndrome components

MetS components	total	IOP < 22	IOP ≥ 22	P^a^
3	2558	2397	161	<0.001
4	823	750	73	<0.001
5	121	110	11	<0.001

Abbreviations: *MetS* metabolic syndrome, *IOP* intraocular pressure

^a^Calculated by chi-square test

P for linear trend < 0.001

Multiple logistic regression analysis adjusted for age and sex, revealed that the presence of more components of MetS had a statistically significant higher risk of increased IOP(*P* < 0.001, Table 3).

**Table 3:** Association of intraocular pressures with metabolic syndrome components

MetS components	Crude OR^a^	P^a^	Adjusted OR^b^	P^b^
non MetS	1.00		1.00	
3	2.21(1.85–2.63)	<0.001	2.16(1.81–2.58)	<0.001
4	3.20(2.49–4.11)	<0.001	3.04(2.35–3.91)	<0.001
5	3.29(1.76–6.13)	<0.001	3.48(1.85–6.53)	<0.001

Abbreviations: *MetS* metabolic syndrome, *OR* odds ratio

^a^Calculated by simple logistic regression analysis

^b^Calculated by multiple logistic regression analysis adjusted for age and sex

In the group with IOP ≥22 mmHg and three components of MetS, the probability of having high IOP was not significantly different compared to the group without MetS when the criteria for HDLC was included, and the combination of BP, TG, and fasting glucose criteria showed the highest odds ratio of 3.73 (*P* < 0.001, Table 4).

**Table 4:** Relationship between intraocular pressure and metabolic parameters in subjects with three components

	IOP < 22	IOP ≥ 22	crude OR^a^	P^a^
non MetS	24,506	746	1.00	
BP + HDL + TG	45	3	2.19 (0.68–7.06)	0.189
BP + HDL + WC	183	9	1.62 (0.82–3.16)	0.163
BP + HDL + DM	7	1	4.69 (0.58–8.19)	0.148
BP + TG + WC	485	35	2.37 (1.67–3.37)	<0.001
BP + TG + DM	97	11	3.73 (1.99–6.98)	<0.001
BP + WC + DM	441	35	2.61 (1.83–3.71)	<0.001
HDL + TG + W	618	23	1.22 (0.38–6.47)	0.351
HDL + TG + DM	42	2	1.56 (0.80–1.89)	0.537
HDL + WC + DM	127	6	1.55 (0.68–-3.53)	0.295
TG + WC + DM	352	36	3.36 (2.37–4.77)	<0.001

Abbreviations: *IOP* intraocular pressure, *OR* odds ratio, *MetS* metabolic syndrome, *BP*, blood pressure, *HDL* high-density lipoprotein cholesterol, *TG* triglyceride, *WC* waist circumference, *DM* diabetes mellitus

Definitions of the metabolic syndrome components were as follows: Fasting glucose ≥ 100 mg/dL (includes diabetes); Blood pressure ≥ 130/85 mmHg or being treated; Waist circumference, women ≥ 85 cm, men ≥ 90 cm; Triglyceride ≥ 150 mg/dL or being treated; HDL cholesterol < 50 mg/dL or being treated

^a^Calculated by simple logistic regression analysis

In the group with IOP ≥22 mmHg and four components of MetS, only the combination of BP, HDLC, abdominal obesity, and fasting glucose criteria showed an increased IOP that was not statistically significant compared to the group without MetS (*P* = 0.550), while the combination of BP, HDLC, TG, and fasting glucose criteria showed the higher odds ratio of 9.39 than of the combination of all five components (*P* < 0.001, Table 5).

**Table 5:** Relationship between intraocular pressure and metabolic parameters in subjects with four or five components

	IOP < 22	IOP ≥ 22	Crude OR^a^	P^a^
non MetS	24,506	746	1.00	
BP + HDL + TG + WC	230	15	2.14 (1.26–3.63)	0.005
BP + HDL + TG + DM	28	8	9.39 (4.26–20.66)	<0.001
BP + HDL + WC + DM	60	1	0.55 (0.08–3.96)	0.550
BP + TG + WC + DM	263	34	4.25 (2.95–6.12)	<0.001
HDL + TG + WC + DM	169	15	2.92 (1.71–4.97)	<0.001
ALL(five components)	110	11	3.29 (1.76–6.13)	<0.001

Abbreviations: *IOP* intraocular pressure, *OR* odds ratio, *MetS* metabolic syndrome, *BP* blood pressure, *HDL* high-density lipoprotein cholesterol, *TG* triglyceride, *WC* waist circumference, *DM* diabetes mellitus

The definitions of the metabolic syndrome components were as follows: Fasting glucose ≥ 100 mg/dL (includes diabetes); Blood pressure ≥ 130/85 mmHg or being treated; Waist circumference, women ≥ 85 cm, men ≥ 90 cm; Triglyceride ≥ 150 mg/dL or being treated; HDL cholesterol < 50 mg/dL or being treated

^a^Calculated by simple logistic regression analysis

## Discussion

Metabolic syndrome is a type of disease cluster in which the primary risk factors for cardiovascular diseases, such as hyperglycemia, hypertension, dyslipidemia, and abdominal obesity, occur simultaneously, and many studies have examined this since the WHO presented the criteria for defining MetS in 1998 [10]. However, even before the concept of MetS was established, studies were conducted on the relationships between factors that comprise MetS and IOP. Many reports have indicated that, in people with obesity, which is one of the components of MetS, the IOP is increased because of a decrease in the aqueous humor outflow due to an increase in orbital fat and episcleral pressure [14–16]. Moreover, high BP increases the pressure in the intraocular ciliary artery, which promotes the formation of the aqueous humor that increases the IOP, and, hence, the association between IOP and hypertension has been confirmed in various studies [16, 17].

Higher serum TG, which is one of the two lipid parameters that comprise MetS, is known to increase the IOP. Klein et al. [18] indicated that, with higher serum TG levels, there is a greater amount of orbital adipose tissue, which causes an increase in orbital pressure, which causes an increase in IOP by inducing an increase in episcleral pressure and a decrease in aqueous humor outflow [19]. Moreover, it has been reported that higher fat intake and an increased severity of obesity results in increased TG levels, whereas HLDC, which is another component of MetS, has a low value, which is the result of increased episcleral pressure and IOP due to an increase in the vascular sclerosing changes and serum osmolality [16].

Hyperglycemia has also been reported to be closely associated with IOP. The continuous fluid inflow into the eyeball from osmotic pressure that is caused by hyperglycemia and the autonomic nervous system dysfunction that is observed in diabetics can induce increased IOP. Additional studies are needed to find out their exact mechanism [20–22].

In addition to the worldwide increase in the obese population, the prevalence of MetS is also showing an increasing trend, and it was reported that 15–25 % of Korean adults suffer from MetS [23, 24]. For this reason, interest in MetS has risen, and many studies on the association between MetS and IOP have been published [25–29]. However, up to now, there have been no reports on the correlation with IOP based on the number and combination of MetS components.

The present study showed that, even within MetS group, the presence of more components of MetS was associated with a higher IOP. As explained earlier, this is because each MetS component is causing factor of increased IOP. In the group with three components of MetS, whitch included the criteria for TG, abdominal obesity, and fasting glucose, the highest risk for IOP increased was shown. In addition, the probability of the IOP increased was significantly higher in the groups that satisfied the criteria for BP, TG, and abdominal obesity; the combination of BP, TG, and fasting glucose criteria; and the combination of BP, abdominal obesity, and fasting glucose criteria.

However, all of the cases that had the HDLC criteria as a component did not have a statistically significant higher risk of IOP. HDLC itself, as shown in Table 1, showed significant differences according to the IOP. However, in the combinations of MetS components, the effect on IOP was relatively limited and was actually influenced more by other factors. Such an interpretation can also be seen in other studies on IOP and lipid parameters, and many reports indicate that serum TG and total cholesterol are positively correlated with IOP, whereas HDLC does not show a corresponding correlation with IOP. High HDLC is negatively correlated with IOP [17, 30, 31], but one report claims a positive correlation with IOP as well [16]. A recent study on Koreans reported a negative correlation between HDLC and IOP in a multiple regression analysis that compensated for age, obesity, and BP [32]. But additional studies appear to be needed to understand the influence of HDLC on IOP in depth.

In groups with four or more components of MetS, the group with BP, HDLC, abdominal obesity, and fasting glucose criteria was the only group that did not show a significant correlation with IOP increase, and, interestingly, compared to the group with all five components, the group with BP, HDLC, TG, and fasting glucose criteria as its components showed the higher risk of IOP increase. However, whether the IOP increases in MetS groups are actually different based on their component combinations is something that requires additional study, as the number of subjects was too small compared to the entire study population when the groups were subdivided according to the MetS components. The genomic variates of subjects could be one of the factors which cause the IOP increases as well [33].

The present study had a few limitations. First, because the present study was based on the regular medical checkup in health promotion center, there was no information of cup to disc ratio and visual field analysis. So it was difficult to investigate the relationship between the glaucoma and metabolic syndrome components with their different combinations. Second, we did not measure central corneal thickness. However, if the IOP that was adjusted according to the central corneal thickness was used, it may have been possible to increase the accuracy. Third, although noncontact tonometers are reliable for testing within normal IOP ranges, the use of a Goldmann applanation tonometer would have given more accurate IOP measurements. Fourth, because the present study was a cross-sectional study, it was difficult to identify the causal relationships. Finally, there was no systemic approach about lifestyle that may have influenced IOP such as smoking history, drinking history, and the frequency of exercise.

Despite these limitations, this study has significance in that it showed not only the fact that each component of MetS is mutually dependent but also the fact that different combination of Mets components has correlation with IOP increase. If additional follow-up studies are conducted, the findings can be used as an indicator for predicting IOP increases in patients with MetS.

## Conclusions

This study showed that there are the differences in the risk of high intraocular pressure according to the different combination of the metabolic syndrome components. In the group with three components of MetS, the combination of BP, TG, and fasting glucose criteria showed the highest risk of intraocular pressure elevation. In the group with four components of MetS, the combination of BP, HDLC, TG, and fasting glucose criteria showed the higher risk of intraocular pressure elevation.

## Abbreviations

BP, blood pressure; DM, diabetes mellitus; HDLC, high-density lipoprotein cholesterol; IOP, intraocular pressure; MetS, metabolic syndrome; TG, triglyceride; WC, waist circumference; WHO, World Health Organization
